# Hepatitis C Elimination During a Global Pandemic: A Case Study of Resilience in Action

**DOI:** 10.1177/00333549221083741

**Published:** 2022-04-09

**Authors:** Shelley N. Facente, Rachel Grinstein, Janessa Broussard, Jessica Shost, Soraya Azari, Jennifer Siruno, Jose A. Jimenez, Anne F. Luetkemeyer, Katie Burk

**Affiliations:** 1 School of Public Health, University of California, Berkeley, Berkeley, CA, USA; 2 Facente Consulting, Richmond, CA, USA; 3 San Francisco Department of Public Health, San Francisco, CA, USA; 4 San Francisco AIDS Foundation, San Francisco, CA, USA; 5 San Francisco Health Plan, San Francisco, CA, USA; 6 University of California, San Francisco, San Francisco, CA, USA; 7 Opiate Treatment Outpatient Program, Zuckerberg San Francisco General Hospital and Trauma Center, San Francisco, CA, USA; 8 Division of HIV, Infectious Diseases and Global Medicine, Zuckerberg San Francisco General Hospital and Trauma Center, San Francisco, CA, USA

**Keywords:** hepatitis C, HCV, elimination, screening, treatment, COVID-19, pandemic, resilience

## Abstract

Until the COVID-19 pandemic, San Francisco’s hepatitis C virus (HCV) elimination initiative, End Hep C SF, was expanding and refining HCV testing and treatment strategies citywide, making progress toward local HCV elimination goals. Although a shelter-in-place health order issued in March 2020 categorized HCV testing as an “essential service,” most HCV testing and treatment immediately stopped until COVID-19–safe protocols could be implemented. During the 14 months of pandemic-related organizational closures, End Hep C SF transitioned to a 100% virtual model, maintaining regularly scheduled meetings. Community-based HCV antibody testing decreased 80% from February to April 2020, and HCV treatment initiation also decreased, although both services started to rebound in mid-to-late 2020, partially as a result of End Hep C SF collaborations. End Hep C SF service providers, clinicians, and advocates reported that the continuous communication and common agenda of End Hep C SF—2 principles of the collective impact initiative—served as a familiar touchpoint and helpful source of information during this isolating and uncertain time. Ultimately, End Hep C SF allowed us to continue HCV elimination strategies through 6 lessons learned: maintaining HCV treatment access through telehealth and mobile services; leveraging research studies that provided HCV testing and treatment; offering HCV screening and linkage to care in tandem with COVID-19–related initiatives; being flexible and inventive, such as administering HCV treatment to residents of shelter-in-place hotels; establishing a data dashboard to track HCV testing and treatment; and relying on partnerships to solve problems and avoid burnout.

Hepatitis C virus (HCV) is a curable infection that has been the number one cause of death in the United States from a nationally notifiable infectious disease since 2013,^1^ until SARS-CoV-2. Due largely to major advances in curative therapy in 2015,^2^ local, national, and international movements for what have become known as hepatitis C elimination strategies have been gaining in popularity. In 2016, the World Health Organization released targets for global HCV elimination by 2030.^3^ The National Academies of Sciences, Engineering, and Medicine followed suit in 2017, identifying national targets for HCV screening and treatment by 2030 as part of the first national hepatitis C elimination strategy.^4^ The push for such strategies on the regional level continued in 2020, when the Centers for Disease Control and Prevention (CDC) released its latest viral hepatitis surveillance funding opportunity, requiring HCV elimination planning as a strategy for any state or local health department that receives funding from 2021 to 2026.^5^

In 2016, San Francisco launched the first city-focused HCV elimination strategy in the United States, with a mission to support all San Franciscans living with and at risk for HCV and to maximize their health and wellness.^6^ As of 2021, more than 190 individuals across 38 organizations had joined End Hep C SF, a multisector consortium independent from the San Francisco Department of Public Health that functions under the principles of collective impact.^7^ Members of End Hep C SF represent syringe services programs, homeless services organizations, pharmacies, private medical systems, grassroots service providers, academia, local government, and a city-focused managed care health plan.^8,9^ Until the COVID-19 pandemic, End Hep C SF was facilitating substantial expansion of HCV testing and treatment strategies throughout the city,^9-12^ making considerable progress toward citywide HCV elimination goals.^13-15^

On March 16, 2020, the San Francisco Health Officer joined 5 other Bay Area counties and issued a shelter-in-place order for all residents of San Francisco in response to the COVID-19 pandemic,^16^ ordering “all businesses and governmental agencies to cease non-essential operations at physical locations in San Francisco” and “directing all individuals living in the county to shelter at their place of residence” except to provide or receive certain essential services.^17^ While the health order allowed HCV-related testing to continue as an “essential service,” both the social distancing requirements and general concern about SARS-CoV-2 transmission had a chilling effect, and most HCV testing and treatment immediately stopped until COVID-19–safe protocols could be implemented. This health order was updated 23 times from March 16, 2020, through May 6, 2021, at which time most businesses were allowed to reopen with limited restrictions as a result of low levels of community transmission and few hospitalizations in the city.^18^

## Purpose

This case study illustrates the impacts of the COVID-19 pandemic on San Francisco’s local HCV elimination efforts and offers lessons learned from our strategies that led us to rapidly restore activities to what are now approaching prepandemic levels. We describe resilience and sustained efforts among our community service providers, clinicians, and advocates and people living with HCV that enabled us to rebuild HCV-related services safely and continue to prevent, diagnose, and treat HCV even during a global public health emergency.

## Methods

During the 14 months of pandemic-related organizational closures, End Hep C SF transitioned to a 100% virtual model, maintaining regularly scheduled meetings using Zoom (Zoom Video Communications). End Hep C SF meetings are planning meetings attended by service providers, policy makers, and community advocates and all members had telephone or computer access allowing virtual participation. By summer 2020, most workgroups had resumed regular meetings even if the agenda included only informal time for sharing the current status of services and lessons learned. Meanwhile, members had previously voted to undertake a new framework to evaluate the initiative’s progress toward HCV elimination, known as Results-Based Accountability (RBA).^19^ Member agencies share aggregated data on their HCV-related activities on a monthly or quarterly basis, which are routinely analyzed and shared with workgroups to help develop interventions and prioritize limited resources during the pandemic.

RBA is especially suited to collective impact work because it examines both individual efforts (ie, those of the individual End Hep C SF partners) and combined efforts. Instead of establishing often arbitrary targets and then striving to meet them in the specified timeframe, an RBA evaluation means determining a series of performance measures that are plotted over time, with the resulting trendlines (known as “the curve”) providing evidence of whether program activities are leading to desired results by turning the curve in the right direction, at maximum possible speed. For End Hep C SF, our performance measures assess the activities that various member agencies are conducting to contribute to our shared goal. For example, one performance measure is the number of community-based HCV antibody tests provided by partners each quarter. Another performance measure is the percentage of tests that were antibody positive. A third measure is the number of treatment starts for people with Medi-Cal (Medicaid) health insurance. Progress is publicly shared via an online data dashboard.^20^

In addition to regular tracking of performance measures via our evaluation dashboard, End Hep C SF conducts a qualitative process evaluation on an annual basis, through workgroup-based focus group discussions and a survey of mostly open-ended questions sent to all members, to gather feedback from people who were not able to attend the focus group sessions. To further explore the impacts of COVID-19 on HCV-related service provision, we asked 4 member organizations to share detailed quarterly data on HCV treatment starts from 2019 through 2020, and we reviewed data on community-based antibody testing routinely submitted by community agencies to the San Francisco Department of Public Health as a condition of program funding. We conducted all data analysis in R version 4.0.5.^21^ As this was an evaluation of routinely collected program data for public health surveillance purposes and did not involve any human subjects research, no approval was sought from an institutional review board for this data review.

## Outcomes

**Figure 1.**
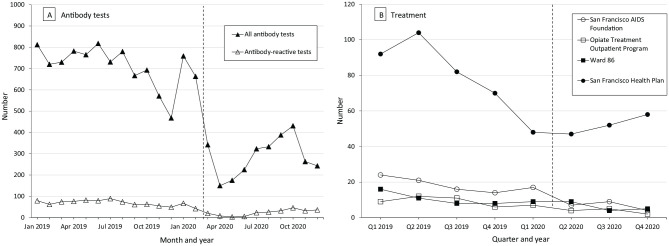
Number of hepatitis C virus (HCV) antibody tests (A) and treatment starts (B), San Francisco, 2019-2020. Abbreviation: Q, quarter. San Francisco Health Plan refers to patients enrolled in the San Francisco Health Plan managed care program, most of whom are MediCal patients. Ward 86 refers to the HIV Clinic at Zuckerberg San Francisco General Hospital. The dashed vertical line indicates COVID-19 shelter-in-place order.^16^

The impact of the COVID-19 pandemic on HCV antibody testing in community venues was profound. The number of testing services decreased as usual from 683 tests in October 2019 to 468 tests during the winter holidays of 2019, rebounded to pre-holiday levels of 759 tests in January 2020 and 663 tests in February 2020, dropped by 48% to only 342 tests in March 2020, and decreased by another 56% to only 150 total citywide tests in April 2020, the first full month after the shelter-in-place order (Figure 1, Panel A). However, thanks in part to ongoing collaboration and shared problem solving during End Hep C SF meetings, community partners were able to resume testing in small numbers in May 2020 and gradually increase services through October 2020 despite a surge in new COVID-19 cases beginning in mid-June 2020, peaking in mid-July 2020, and tapering off through October 2020. In October 2020, the number of HCV antibody tests decreased, this time also corresponding with a third local spike in COVID-19 cases.^22^

We found a similar pattern in HCV treatment starts, although the effect of COVID-19 was less pronounced than with testing services (Figure 1, Panel B**)**. We included in our analysis the number of people initiating HCV treatment in 4 settings—primary or specialty care for Medi-Cal patients served by the San Francisco Health Plan, the San Francisco AIDS Foundation’s syringe service program or sexual health clinic, the hepatitis co-infection clinic based at the HIV clinic in Zuckerberg San Francisco General Hospital and Trauma Center (ZSFG), and the Opiate Treatment Outpatient Program, a methadone clinic at ZSFG. Although early treatment initiation efforts at these sites resulted in many patients treated during 2016-2018, by quarter 3 of 2019, the number of patients initiating treatment had begun to decline, as patients most easily treated had already been cured. Most patients remaining to be treated in these locations by the time the COVID-19 pandemic hit required extra supportive structures to initiate treatment; nonetheless, with the support of End Hep C SF, treatment numbers began to rebound during the pandemic recovery period beginning in quarter 3 of 2020. While standard telehealth visits proved challenging for most patients, ZSFG simplified the treatment initiation process during COVID-19 by having HCV specialists provide advice remotely to primary care providers or hospital inpatient teams, without directly seeing the patient. Given that many HIV/HCV co-infected patients take medications with overlapping toxicities, a week 4 liver function/renal panel was still standard practice; however, HCV RNA was only checked 12 weeks post–treatment completion, thus simplifying laboratory processes. The ZSFG pharmacy and specialty pharmacies were also critical to treatment success during COVID-19. Local specialty pharmacies arranged for home delivery of medications to patients—critical during a time when patients were limiting public interactions for safety reasons—and were able to notify the ordering clinician whenever medications were interrupted.

**Figure 2.**
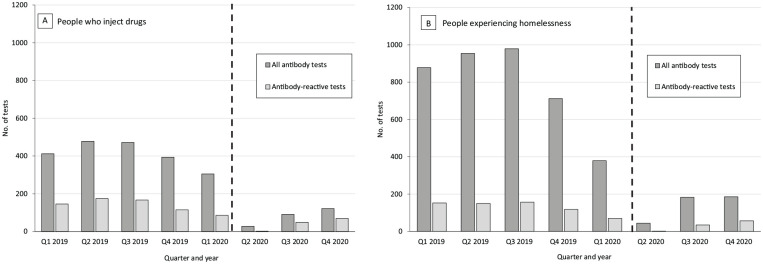
Number of community-based antibody tests, by quarter (Q), San Francisco, 2019-2020. The dashed vertical line indicates COVID-19 shelter-in-place order.16 Antibody-reactive tests indicate past or present HCV infection.

We also examined the pre- and peri-pandemic patterns of community-based antibody testing among the high-prevalence populations most underserved by traditional services: people who inject drugs (PWID) (Figure 2, Panel A) and people experiencing homelessness (PEH) (Figure 2, Panel B). In 2019, a high proportion of community-based HCV antibody tests was conducted among these populations because of targeting strategies of testing and outreach programs among End Hep C SF member organizations. In quarter 2 of 2019, 20.2% (n = 478) of 2365 total tests were among PWID and 40.3% (n = 954) of total tests were among PEH. In quarter 2 of 2020, only 5.1% (n = 28) of 550 total tests were among PWID and 8.0% (n = 44) were among PEH, illustrating that those who were able to access antibody testing during the early phases of the pandemic response tended to be those more typically well-served by social systems. We found no meaningful trends in the demographic characteristics of people testing for HCV antibodies or initiating HCV treatment by quarter during 2019-2020.

During the qualitative portion of End Hep C SF’s process evaluation in 2020, members of End Hep C SF identified the initiative’s continued virtual operation during the pandemic as a major source of information and momentum to resume services. The initiative’s sustained operation was especially helpful under the principles of collective impact, where members noted that the common agenda of HCV-related services to eliminate HCV was a valuable piece of non–COVID-19–related familiarity among the pandemic uncertainty. Similarly, they recognized that continuous (virtual) communications served as a critical touchpoint for people who were physically isolated, during a time when workplace communications were otherwise severely disrupted.

## Lessons Learned

People at risk for HCV infection and complications from untreated chronic HCV continue to be at risk during the COVID-19 pandemic. In fact, PWID and PEH may have been at even greater risk during early phases of the pandemic, at least in part because of forced isolation and a reduction in supportive services. In San Francisco, the impact of increased isolation has been acutely visible through fatal drug overdoses: 713 people died of an accidental drug overdose in San Francisco in 2020, a 61% increase from 2019, despite a robust citywide naloxone training and distribution program.^23^ Furthermore, the same people at highest risk for HCV infection and complications are the same people most impacted by COVID-19. PEH compose 0.91% of the city’s residents yet 1.94% of reported SARS-CoV-2 cases.^22^ One notable COVID-19 outbreak occurred in a major city-run homeless shelter in April 2020,^24^ when 11.5% of the city’s monthly cases were among PEH.^22^ While limited data are available on COVID-19 among PWID, many PWID live in single-room-occupancy (SRO) hotels; as of April 30, 2021, 1654 residents across 304 SRO buildings in San Francisco had received a positive test result for SARS-CoV-2, including 412 people in city-run shelter-in-place sites set up as alternative housing for PEH during the pandemic.^25^ Taken together, these statistics highlight the critical importance of continuing to sustain HCV prevention, testing, and treatment efforts during a pandemic, which our End Hep C SF structure enabled. Furthermore, given the overlapping factors that make people vulnerable to both HCV infection and COVID-19, strategies designed to prevent one disease frequently improve prevention for the other disease at the same time.

Many End Hep C SF members were resilient, passionate, and creative about continuing to help people living with or at risk for HCV infection. Monthly meetings were an invaluable space for frontline workers to share ideas and information on how to safely scale up HCV services in later 2020, rather than relying on the overburdened health department to issue sector-specific guidance.

To aid public health workers throughout the United States and internationally in continuing efforts to reduce HCV-related morbidity during a public health emergency, we present 6 lessons learned from our experience that have helped us continue our HCV elimination strategies despite the COVID-19 threat.

1. We maintained HCV treatment access, albeit at reduced rates, through sites providing essential services. Often, services were offered via outreach workers or medical assistants working in a van where a prescribing clinician could be reached via telehealth as needed for higher-level consultation or medication prescription.
2. We leveraged research studies providing HCV testing and treatment wherever possible, as studies funded by the National Institutes of Health or pharmaceutical foundations were often more easily able to pivot and safely enroll people as participants, whereas participants might otherwise fall through the cracks of a community organization under staffing furloughs during COVID-19. For example, we were able to connect many of our community (peer) navigators to a local research study offering HCV treatment on demand, which allowed them to safely practice their navigation and peer support skills and link people to treatment even when the official community navigator program was suspended because of COVID-19. If other jurisdictions do not have operational research studies, settings such as syringe service programs or homeless services could be similarly leveraged to continue offering HCV testing and treatment to those most in need when regular HCV services are temporarily stopped.
3. We offered HCV screening and linkage to care alongside COVID-19–related initiatives where possible. Offering these services was not practical at mass COVID-19 testing or vaccination sites but was realistic for community organizations to provide in SROs and shelter-in-place hotels for PEH, in partnership with the city health department. COVID-19 testing and vaccination were also prioritized for communities highly impacted by HCV, in collaboration with organizations already serving them. In 2021, the city health department began to offer COVID-19 vaccines at HCV testing sites and began to allow HCV and HIV testing at smaller niche sites offering COVID-19 vaccines.
4. We were flexible and inventive, looking for silver linings. For example, San Francisco’s unprecedented effort to provide longer-term shelter for PEH during COVID-19 through shelter-in-place hotels led to an opportunity for End Hep C SF to partner with city departments to initiate HCV treatment while hotel residents had stable housing—including a place to safely store medications—for the first time in years.
5. The public demand for near–real-time COVID-19 data led to the rapid establishment of a data dashboard hosted by a government entity in San Francisco known as Data SF.^25^ End Hep C SF is working with Data SF to improve the use and visibility of HCV data moving forward. This process was also a catalyst for our own established data dashboard,^20^ which was built by a consultant paid by End Hep C SF, using widely available, user-friendly Clear Impact software (ClearImpact LLC). This dashboard also highlights the importance of not only plotting the data of measures at each timepoint but also holding regular discussions to understand the story behind the curve. For example, we knew our testing and treatment numbers were substantially lower in the middle quarters of 2020 than in the last quarter of the year. Through workgroup discussions, we were able to understand more about why we saw decreasing treatment initiation numbers even pre–COVID-19, in 2019.
6. Finally, we learned the value of partnerships in times of crisis. Our initial instincts were to cancel End Hep C SF meetings because community service providers, clinicians, and advocates were too busy or overwhelmed dealing with the COVID-19 emergency to attend HCV elimination meetings. However, as the pandemic dragged on, it became clear that regular connection through End Hep C SF meetings was a lifeline for End Hep C SF members. Lacking clear guidelines on personal protective equipment and COVID-19 testing when working in the field, our partners consulted each other for information and ideas to keep themselves safe. When one community organization was able to begin HCV testing in shelter-in-place hotels, it coordinated with a mobile van known as DeLIVER Care to park outside during HCV testing hours, so that people who received a positive test result could go straight outside to the van to receive confirmatory HCV RNA testing and, if positive, begin treatment through the van.^26^

In summary, the resilience, creativity, and ongoing commitment of our End Hep C SF providers allowed us to sustain progress toward HCV elimination during a global pandemic. Any jurisdiction—including jurisdictions that are resource-constrained—can take steps to prevent or treat HCV by leveraging existing services and problem solving collaboratively as the local situation evolves. As budget constraints threaten deep cuts in nonessential services, it will be doubly important to preserve programs designed to reach residents who are most vulnerable to both HCV and COVID-19 and integrate the ongoing threat of COVID-19 into regular services. In San Francisco, we are confident that our community will continue to drive toward our elimination goals.
